# CST: A Multitask Learning Framework for Colorectal Cancer Region Mining Based on Transformer

**DOI:** 10.1155/2021/6207964

**Published:** 2021-10-11

**Authors:** Dong Sui, Kang Zhang, Weifeng Liu, Jing Chen, Xiaoxuan Ma, Zhaofeng Tian

**Affiliations:** ^1^School of Electrical and Information Engineering, Beijing University of Civil Engineering and Architecture, Beijing 100044, China; ^2^Department of Laboratory and Diagnosis, Changhai Hospital, Navy Medical University, Shanghai 200433, China

## Abstract

Colorectal cancer is a high death rate cancer until now; from the clinical view, the diagnosis of the tumour region is critical for the doctors. But with data accumulation, this task takes lots of time and labor with large variances between different doctors. With the development of computer vision, detection and segmentation of the colorectal cancer region from CT or MRI image series are a great challenge in the past decades, and there still have great demands on automatic diagnosis. In this paper, we proposed a novel transfer learning protocol, called CST, that is, a union framework for colorectal cancer region detection and segmentation task based on the transformer model, which effectively constructs the cancer region detection and its segmentation jointly. To make a higher detection accuracy, we incorporate an autoencoder-based image-level decision approach that leverages the image-level decision of a cancer slice. We also compared our framework with one-stage and two-stage object detection methods; the results show that our proposed method achieves better results on detection and segmentation tasks. And this proposed framework will give another pathway for colorectal cancer screen by way of artificial intelligence.

## 1. Introduction

Colorectal cancer is a common malignancy tumour worldwide, which has ranked the third position as the most common cancer and the second cause of cancer-related deaths worldwide. Also, the 2021 analysis observes that the diagnosed patients are rising in the crowd younger than 50 years old [1]. In China, there are more than 480,000 new cases with a higher than 30% death percentage in 2020, which increases the incidence and mortality rates rank following lung cancer [2]. In the early stages, occult blood examination and medical images were employed for clinical detection and diagnosis. These methods exhibited a productive approach for the early colorectal cancer diagnosis and can improve the survival of these patients [3]. However, mere blood examination and colonoscopy inspection could not reveal the biological morphology and tumour statutes [4, 5]. In the past decades, imaging approaches such as computed tomography (CT) and magnetic resonance imaging (MRI) have become an effective way for colorectal cancer diagnosis; doctors can get an overall scheme of the tumour region in a comprehensive way without invasion [2, 6]. In addition, medical imaging can get detailed information of the tumour region without any physical cathartic cleansing, which has become a prevalent screening guideline [7, 8].

Medical image processing and analysis have achieved remarkable progress in the past several years, especially the use of deep learning, but this field is still challenging for the difficulty of acquisition and annotation in medical imaging datasets [9, 10]. In this context, transfer learning is another pathway for handling the lack of annotated medical data with small-scale data training and becomes a common protocol instead of traditional supervised learning [9, 10]. As an effective way, the transfer learning protocol in medical image processing usually employed the ImageNet pretrained deep architecture, *e.g.*, ResNet, DenseNet, FCN, and U-Net family, and then, these models are fine-tuned on small-scale medical images to fit some certain tasks [11–15]. These fine-tuned models yield much better results than bottom-up training strategy, especially when it confronts a small set of image samples.

However, despite 3D or 2D medicine having the same image structures, it is worth emphasizing that especially in tasks of computer vision, these medical images have distinct interest with the natural image benchmark datasets. In the case of medical data, most of the ROIs took part in a small region in the whole image, resulting in sophisticated yet hard example problems [16, 17]. Learning a medical image analysis network transferred from the natural scene usually leads to strong bias without considering the characteristic of the medical image. Thus, all of the above formations motivate developing a transfer learning protocol directly by a medical imaging dataset that can handle subtle variances in these two datasets. In this situation, ConvNets become a popular backbone in many medical image and nature image processing protocols. But for the 3D image tasks, the traditional ConvNets cannot perform long-range dependency modelling. In addition, applications of pyramid ConvNets and some attention mechanisms can facilitate the processing of these sequential image datasets [18]. However, most of these methods have not focused on long-range medical image pipeline on multitask tumour region analysis.

Transformer models such as BERT and DERT have successfully achieved the state of the art in nature language processing and computer vision fields [19–22]. Due to its ability to learn long-range dependencies from input tokens, the self-attention mechanism can model the dependency among the input tokens. The famous vision transformer (ViT) have achieved comparable performance with the traditional deep learning model such as the CNN model on image recognition tasks [23]. But all of these models have to be trained on a large-scale dataset. DeiT (Data-efficient Image Transformers) is the first transformer-based model adapted by midsized datasets [24].

In this work, we propose a novel transfer learning protocol, called *CST*, which is a union framework for colorectal cancer region detection and segmentation task based on the transformer model, which effectively constructs the cancer region detection and its segmentation jointly. To make a higher detection accuracy, we incorporate an autoencoder-based image-level decision approach that leverages the image-level decision of a cancer slice. First of all, we pretrain an encoder-decoder architecture for cancer/normal image slice representation that generates the encoding vectors of the original input image slices as the image-level label. Then, another transformer-based global to local architecture is pretrained by our colorectal medical image datasets for tumour region detection and segmentation. To validate the effectiveness of our proposed framework, we test the model output on the collected colorectal cancer MRI image series and achieved remarkable performances compared with other traditional methods.

In summary, our main contributions are as follows:

- We propose a novel framework for colorectal cancer region detection and segmentation. Our framework provides a more flexible pathway for tumour region mining
- We combine the traditional autoencoder and transformer architecture together for the multitask framework for the final decision
- We evaluate the proposed method on the colorectal cancer MRI image dataset, and our method has achieved a better result on tumour region detection and segmentation

## 2. Related Works

Convolution neural networks with their excellent feature representation ability have raised a revolution in the nature language processing field, as well as the computer vision and signal processing fields [25]. Position-sensitive tasks such as semantic segmentation that contains several parts of ROIs have been well represented by using convolutional encoder-decoder architectures [26, 27]. The main aim of convolutional operation is to catch local texture feature information by the convolution kernel, and more layers and stride kernel in the receptive field can extend the capture range during downsampling; in this way, the model can capture global to local information explicitly. However, the size and shape of these kernels are usually of fixed size and cannot adapt to all the input range [28–30].

Recent advance in transformer-based architecture with a self-attention mechanism and the ability of long-range modelling has achieved the state of the art in natural language processing and computer vision [31]. Vision transformer (ViT) can treat the whole image into several patches and feed into the transformer pipeline as tokens. The simple application of the transformer has shown excellent results compared with the traditional CNN model [23]. However, the computation cost and large-scale dataset are the fatal drawbacks to competing with the convolutional neuronal network.

In the medical image processing paradigm, few annotated clinical data cannot generate efficient models and have to use the ImageNet pretrained model for the downstream tasks [32]. In practice, most of the prevalent methods use their pretrained weights for the medical ROI detection, such as ResNet and DenseNet, and fine-tune the higher layers on some special tasks. But most of these strategies is limited to applying for new datasets [11–15]. In a word, the pretrained model on ImageNet and other datasets by fully supervised learning paradigm have to be severed with massive annotated datasets to fit the downstream transfer learning. In another way, a self-supervised learning framework can get a suitable result by using few or no need of labelled datasets; this has gained great attention in medical image analysis recently [33, 34]. Furthermore, the self-supervised learning paradigm has attracted great attention in the medical image analysis field [35]. The critical challenge for self-supervised learning is how to define a suitable proxy task from the unlabelled data. But most of these proxy tasks have exhibited less use on medical image-related tasks.

## 3. Material and Methods

### 3.1. Dataset

**Figure 1:**
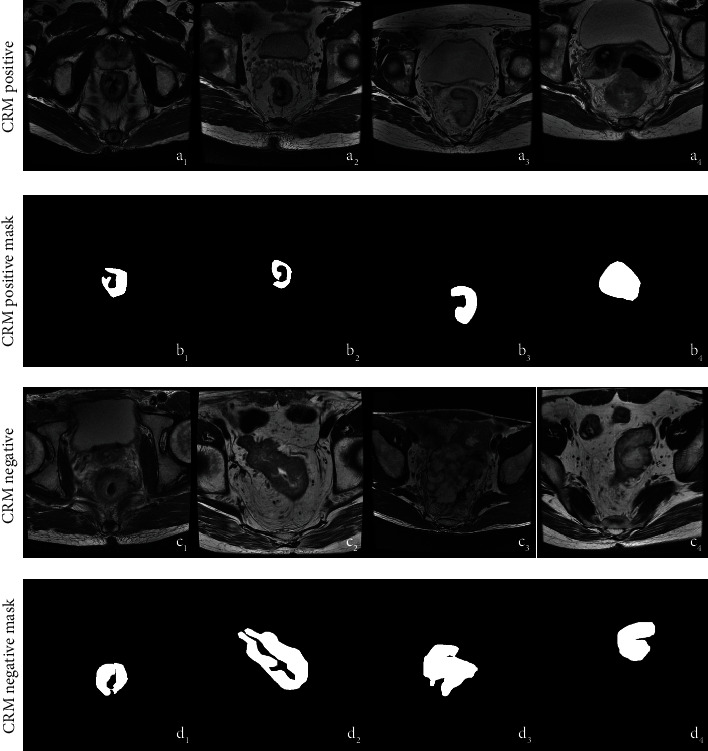
Examples of colorectal cancer in MRI images. (a1–a4, c1–c4) Are the original image slices from the MRI DICOM series; (b1–b4, d1–d4) are the tumour region mask labelled by doctors.

We construct a novel dataset for this research, which contains 375 cases of colorectal cancer tumour MRI image datasets from 2013 to 2020, which contains 289 CRM negatives and 86 CRM positives. For segmentation and detection tasks, we also collect 375 cases of colorectal cancer negative samples as the negative samples for deep architecture training. Our collaborator labels the image slices with mask and bounding box separately as the final ground truth. Then, the dataset was divided into training, testing, and validation sets for the network training and evaluation. The main aims of this dataset are to collect for colorectal cancer region detection and segmentation, and to follow this aim, we construct the framework in this manuscript to perform them and prepared for the clinical applications.  Figure 1 shows the details of the labelled tumour region about CRM negative and positive, respectively.

### 3.2. Multitask Framework

**Figure 2:**
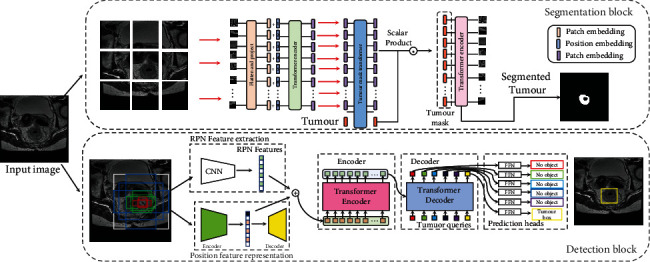
Schematic diagram of the proposed multitask learning framework for colorectal cancer region mining frame.

The motivation of our work is to construct a multitask framework that combines tumour region detection and segmentation tasks. In this section, we illustrate the overall framework of our proposed CST framework as shown in Figure 2. Our framework is divided into two pipelines, the tumour region detector and the tumour segmentation pipeline. In the detection pipeline, we first generate the region proposal of the input images, and an encoder-decoder model is used for the position encoder as the DETR input. In the segmentation pipeline, we use image patches as the input and project to a sequence of embedding for the transformer, and the class embedding is used for the final mask prediction.

#### 3.2.1. Detector Pipeline

In this part, we start from the region proposals generated from an input medical image with *H* and *W* in height and width as the initial image *x*_rpn_ ∈ ℝ^3×*H*_0_×*W*_0_^ with 3 channels, *H*_0_ in height and *W*_0_ width of the RPN. We choose a conventional CNN backbone to generate the lower resolution activation map *f* ∈ ℝ^*C*×*H*×*W*^; typically, the values of *C* = 2048; *H*′ and *W*′ are resized as the initial input of the setting *H*′ = *H*/32, *W*′ = *W*/32. Unfortunately, the position encoding method in the original image only reflects the location of the pixels in the column and row, but the input position in our pipeline is from the random selected RPNs, so in this part, we pretrained an autoencoder for the position representation and we added this coding to the traditional position encoding with the anchor position together.

For the transformer encoder, we use a 1 × 1 convolution to reduce the dimension of the activation map into a *d*-dimension vector; for the input of the transformer, the feature map is collapsed into a 1-dimension vector with a *d* × *HW* feature map. Each encoder layer is adopting the standard setting as is stated in the DETR [19–22]. For the transformer decoder, it follows the standard architecture of the transformer, and the model can decode the tumour region at each decoder layer. Each object/RPN is transformed into an output embedding by the decoder. They are decoded into bounding box coordinates and tumour/nontumour class labels by the following feed forward network (FFN). The FFN is a 3-layer perceptron with ReLU, hidden dimension *d*, and a linear projection layer. It can predict the normalized centre, height, and width of the tumour bounding box. In addition, the tumour and nontumour class of the detected bounding box is predicted by a SoftMax function.

The loss function of the tumour detection part is to optimize the lowest cost:

(1) $$\begin{array}{c} \hat{\sigma }=\underset{\sigma \in {℘}_{N}}{\arg\min}\overset{N}{\underset{i}{\sum }}L_{\text{match}}\left(y_{i},{\hat{y}}_{\sigma \left(i\right)}\right), \end{array}$$

(2) $$\begin{array}{c} L_{\text{match}}\left(y_{i},{\hat{y}}_{\sigma \left(i\right)}\right)=L_{\text{box}}\left(b_{i},{\hat{b}}_{\sigma \left(i\right)}\right)-{\hat{p}}_{\sigma \left(i\right)}\left(c_{i}\right). \end{array}$$

For efficient computing, we choose optimal assignment with the Hungarian algorithm to accelerate the training process. Here, we use Hungarian loss for all pairs matched, and the object detector loss is defined like similarly loss; the total Hungarian loss is defined as follows:

(3) $$\begin{array}{c} L_{\text{Hungarian}}\left(y,\hat{y}\right)=\overset{N}{\underset{i=1}{\sum }}\left[-\log{\hat{p}}_{\hat{\sigma }\left(i\right)}\left(c_{i}\right)+L_{\text{box}}\left(b_{i},{\hat{b}}_{\hat{\sigma }}\left(i\right)\right)\right]. \end{array}$$

(4) $$\begin{array}{c} L_{\text{box}}\left(b_{i},{\hat{b}}_{\hat{\sigma }}\left(i\right)\right)=\lambda_{\text{iou}}L_{\text{iou}}\left(b_{i},{\hat{b}}_{\sigma \left(i\right)}\right)+\lambda_{L1}{\left\|b_{i}-{\hat{b}}_{\sigma \left(i\right)}\right\|}_{1}, \end{array}$$

where for the prediction with index *σ*(*i*), *y*_*i*_ = (*c*_*i*_, *b*_*i*_) is the labelled ground truth, and we define the probability of the tumour region; we define *c*_*i*_ as ${\hat{p}}_{\hat{\sigma }\left(i\right)}\left(c_{i}\right)$ and the predicted bounding box as ${\hat{b}}_{\sigma \left(i\right)}$, *λ*_iou_ ∈ ℝ and ℝ_*L*1_ ∈ ℝ.

#### 3.2.2. Segmenter Pipeline

The segmenter part is based on full transformer-based architecture for pixel-level class annotation. As shown in the upper part of Figure 2, we model the sequence of patches by using a transformer encoder and a point-wise linear mapping or a mask transformer. The whole pipeline is trained end-to-end with cross-entropy loss per pixel.

In the encoder part, we first split the input image into a sequence of identical size patches, and each patch is flattened into a 1-dimension vector to produce a sequence of patch embeddings. For the position information encoding, we treat each patch as a separated part from the whole image and finally add position information to the original patch position. After that, the traditional transformer encoder is employed for the sequential information encoding with a multihead self-attention block. For the decoder part, it first learns to map patch-level encodings from the encoder to patch-level class scores; following that, these scores are unsampled to pixel-level scores by bilinear interpolation. The whole mask transformer is illustrated in the lower part of Figure 2.

For the mask transformer, we use a set of *K* learnable class embeddings in the decoder; in our pipeline, *K* is 2. Each class embedding is randomly initialized and assigned to a single class so as to generate the class mask. At last, the class embeddings are processed jointly with patch encodings by the decoder as depicted in Figure 2. The total loss function is defined as follows:

(5) $$\begin{array}{c} L_{\text{tumour}}=-\overset{n}{\underset{i=1}{\sum }}t_{i}\log\left(p_{i}\right),\;\text{for }k\text{ classes}, \end{array}$$

where *t*_*i*_ is the ground truth label and *p*_*i*_ is the softmax probability for the *i*^th^ class.

In this way, we combine these loss functions to form an end-to-end train protocol, for the total loss is defined as follows:

(6) $$\begin{array}{c} L_{\text{total}}=L_{\text{match}}+L_{\text{tumour}}. \end{array}$$

Our proposed method employs a simple process to treat the patch and tumour region segmentation jointly during the decoding phase in the segmentator; in the whole framework, we address tumour region detection and segmentation jointly and combine them into a whole framework.

## 4. Results and Discussion

### 4.1. Implementation Details

In the tumour region detection pipeline, we train DETR with AdamW optimizer with the initial transformer's learning rate to 10^−4^, the backbone's to 10^−5^, and the weight decay to 10^−4^. We choose the transformer weights with Xavier in it and all backbone is ImageNet pretrained ResNet50 model for the basic architecture [36, 37].

In the tumour segmentation pipeline, the architecture is based on the vision transformer (ViT), and the head size of the multihead self-attention block is fixed to 64, other parameters are set as the default of the ViT model, and the input patches are with the same size [23, 24]. The segmentation model is pretrained on ImageNet; ViT is pretrained on ImageNet with random cropping. Following that, we fine-tune the pretrained models for the tumour region segmentation task and the pixel-wise cross-entropy loss without weight rebalancing. In the training phase, the SGD optimizer with a base learning rate 0.0001 and weight decay 0 is set in the initial training paradigm.

Here, we choose the standard evaluation method of tumour detection and segmentation. The Jaccard index is used for evaluating the ground truth bounding box and the predicted bounding box variances, and formally, the IoU measures the overlap between the ground truth box and the predicted box over their union. The total IoU is defined as follows:

(7) $$\begin{array}{c} {\text{IoU}}_{\text{pred}}^{\text{truth}}=\frac{\text{truth}\cap \text{pred}}{\text{truth}\cup \text{pred}}. \end{array}$$

For comparisons with other methods, the results of our framework and other methods are reported in terms of recall, precision, and f1-measure values as follows:

(8) $$\begin{array}{c} \text{Recall}=\frac{\text{TP}}{\text{TP}+\text{FN}}, \end{array}$$

(9) $$\begin{array}{c} \text{Precision}=\frac{\text{TP}}{\text{TP}+\text{FP}}, \end{array}$$

(10) $$\begin{array}{c} \text{F}1=\frac{2PR}{P+R}. \end{array}$$

Our framework is performed on the environment of Ubuntu 14.04 with Inter Core i9 platform, 32 GB RAM, and 2080Ti GPU×2 and is based on the PyTorch platform, CUDA v10.0, cuDNN v8.0.

### 4.2. Experiment Results

For the model training, we first divide the two pipelines separated, and training them individually, it takes about 72 hours and 103 hours for the detector and segmentator. For the restriction of our GPUs, we have not extended other comparisons with other transformer-based methods.

**Figure 3:**
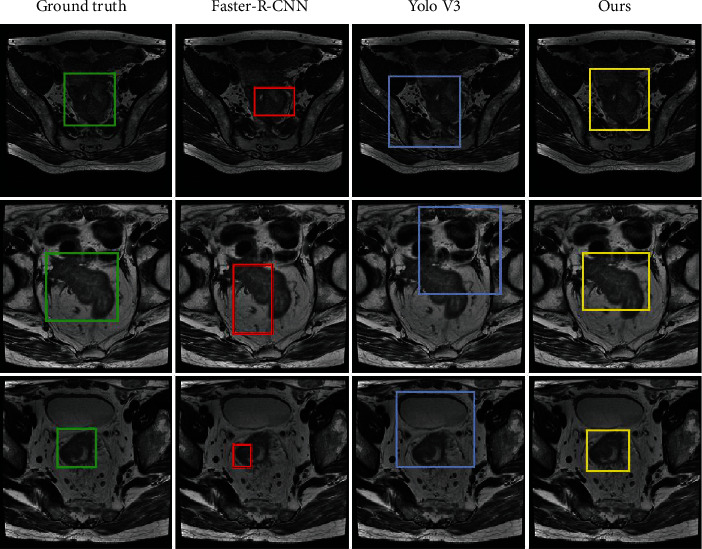
Tumour region detection results and its comparison results.

For tumour detection, we divide the dataset into training and validation sets. For the same baseline, these methods cover the one-stage and two-stage object detection pipelines. The most prevalent method Faster-RCNN and Yolo-V3 are chosen as the test bed for the final comparisons.  Figure 3 shows the detection results of the three methods, our proposed pipeline can cover most of the tumour regions, and the bounding box can converge to the tumour boundary accurately. The Faster-RCNN model is a popular two-stage object detection method, which can catch the tumour region, but it is affected by the changes of the background; this is largely because the tumour regions have the same texture as other organs. The Yolo-v3 is a popular two-stage object detection method and has been employed for many object detection and location tasks. We have evaluated this method on this dataset, the results are shown in column 3 of Figure 3, it shows that Yolo-v3 has detected the tumour region, but the result exhibits that this method usually covers the tumour region and the neighbour organs together and this is largely affected by the distinct boundary of these regions.

**Figure 4:**
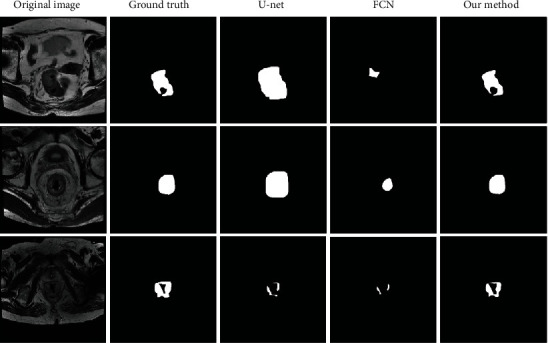
Basic rocket ship design. The rocket ship is propelled with three thrusters and features a single viewing window. The nose cone is detachable upon impact.

For the tumour segmentation, we have the same separation of dataset like the detection pipeline. We set the U-Net and FCN as the baseline for the comparison, the U-Net is a specific method for medical image analysis, and FCN have been greatly used in the nature image segmentation; for this reason, we have listed the comparison result with these two methods and to exhibit our framework's advantages. In this part, the U-Net model has better results than the FCN model, and it can catch the tumour region in high contract images but less in low contract slices. The FCN model usually needs an intensive training protocol on large image data, but in this program, the dataset is less than those, so it cannot get better results. Compared with these two methods, our proposed method has achieved an excellent result on the image segmentation tasks. And the results are shown in Figure 4.

For a better comparison, we also compared the accuracy of these two methods, and our proposed framework also achieves a better result on the dataset (see Tables 1 and 2).

## 5. Conclusions

In this paper, we propose a novel transfer learning framework, CST. We combine the colorectal cancer region detection and segmentation task jointly and fine-tuned a transformer-based model to perform these tasks. For higher accuracy, we incorporate image-level information into the final cancer region detection, the results demonstrate that the proposed framework can handle these tasks well, and the comparison results have shown that our method has achieved better accuracy than the traditional methods such as CNN. In this way, the proposed framework explores a new protocol for colorectal cancer information mining. In future works, we mainly focus on how to use few samples to achieve a better result.

## Figures and Tables

**Table 1:** Tumour region detection results covered by the study.

Methods	CRM+ (%)	CRM- (%)	Average (%)	Total (%)
Faster-RCNN	67.1	62.3	64.7	65.6
Yolo-v3	43.4	37.6	40.5	41.2
Ours	87.5	89.1	88.3	88.6

**Table 2:** Tumour region segmentation accuracy covered by the study.

Methods	CRM+ (%)	CRM- (%)	Average (%)	Total (%)
U-Net	82.1	81.7	81.9	81.8
FCN	67.2	66.4	66.8	66.5
Ours	91.2	90.6	90.9	91.1

## Data Availability

The DICOM data used to support the findings of this study were supplied by Zhaofeng Tian and Jing Chen under license and so cannot be made freely available. Requests for access to these data should be made to Dong Sui.
